# Differential DNA Extraction from Lateral Flow Immunochromatographic Tests via the EZ1^®^ Advanced XL System

**DOI:** 10.3390/mps8010002

**Published:** 2025-01-02

**Authors:** Scarlet Neilson, Leah Nangeroni, Mirna Ghemrawi

**Affiliations:** 1 Department of Chemistry & Physics, Arcadia University, 450 S Easton Rd, Glenside, PA 19038, USA; scarlet.neilson@gmail.com; 2 The Center for Forensic Science Research and Education, 206 Welsh Road, Horsham, PA 19440, USA; mirna.ghemrawi@cfsre.org

**Keywords:** lateral flow immunochromatographic (LFI) tests, body fluid identification, differential DNA extraction, body fluid mixtures

## Abstract

This differential extraction protocol details the steps for isolating DNA from sample pads used in lateral flow immunochromatographic (LFI) tests, particularly for cases involving mixed biological samples such as semen and menstrual blood, or other evidence related to sexual assault. This procedure utilizes a differential extraction technique applied to sample pads from immunochromatographic tests, where the sample pads serve as the substrate. The method involves two sequential lysis steps to effectively separate non-sperm and sperm fractions, enabling the targeted isolation of distinct cell types for downstream DNA analysis. The efficiency of this procedure is demonstrated by the results within this paper, which highlights the successful recovery of both male autosomal and Y-STR profiles, even in mixed samples with a high female presence. Overall, this protocol demonstrates the effective recovery of DNA from sample pads, which is beneficial for forensic practitioners dealing with limited sample quantities, underscoring the value of using these pads in forensic analysis.

## 1. Introduction

A common technique to identify the possible presence of bodily fluids found at a crime scene is utilizing lateral flow immunochromatographic (LFI) assays. In LFI assays, a sample that contains the antigen of interest moves up the test membrane through capillary action, passing through test zones where antibodies (generally IgG) are located [1]. Antigens of interest will bind to these antibodies, resulting in a visible test result line. The SERATEC^®^ PMB Test identifies possible peripheral blood and/or menstrual blood in forensic samples by rapidly detecting human hemoglobin and human D-dimer, respectively [2]. The SERATEC^®^ PSA Semiquant is used to identify the possible presence of semen through the detection of prostate specific antigen (PSA) in forensic samples [3]. Following serological testing, DNA extraction—often using differential extraction in cases of sexual assault—is performed, followed by DNA typing. This process is particularly valuable in the context of sexual assault cases for analyzing DNA profiles from mixed biological samples. The sample pads from these tests can be further utilized and directly extracted from, minimizing the consumption of critical evidence. Additionally, other fields that utilize LFI tests in other applications could also use these pads for further analysis.

Differential extraction is an extraction method to separate sperm cells that may be present in a sample from other cells (such as epithelial cells) [4]. This method is often used when processing sexual assault cases to separate non-epithelial and epithelial cells in a sample, which can aid in mixture interpretation during analysis [4]. The non-sperm cells are first lysed and then pelleted to remove the supernatant (fraction 1). The pellet is then washed before going through a more stringent lysis step, which uses dithiothreitol (DTT) to reduce the strong disulfide bonds, ultimately resulting in the “sperm fraction” (fraction 2). These fractions can be further purified, resulting in extracts of purified DNA from the non-sperm cells (fraction 1) and from the sperm cells (fraction 2).

Extracting directly off the sample pad of immunochromatographic tests can be a particularly useful method, especially in situations where initial evidence is limited, or where the entire volume available was used for testing on the immunochromatographic assay [5,6,7,8,9]. According to previously published studies, there are no known interactions with the immunochromatographic buffers and the extraction reagents [5,6]. The previously published literature described extraction from sample pads utilizing manual extraction methods, and an automated extraction method using the Maxwell instrument [5,8,9]. None of these publications attempted to perform differential extraction from LFI pads. Therefore, the following protocol describes a semi-automated differential extraction protocol from immunochromatographic sample pads utilizing the EZ1^®^ Advanced XL Instrument, which results in purified DNA fractions that can be utilized for downstream processing. Using these sample pads as the substrate with the differential extraction method can prove resourceful, especially for sexual assault cases, where biological samples are frequently mixed, and original evidence may be limited.

## 2. Experimental Design

### 2.1. Reagents and Materials

- SERATEC^®^ PMB Test Kit (SERATEC GmbH, Göttingen, Germany);
- SERATEC^®^ PSA Semiquant Kit (SERATEC GmbH, Göttingen, Germany);
- EZ1&2^®^ Advanced XL DNA Investigator^®^ Kit (QIAGEN, Hilden, Germany);
- 1 M Dithiothreitol Solution (DTT) (Thermo Fisher Scientific, Waltham, MA, USA);
- Sterile pipette tips (2.5 µL, 10 µL, 20 µL, 100 µL, 200 µL, and/or 1000 µL) (Neptune Scientific, San Diego, CA, USA);
- 2 mL microcentrifuge tubes (Eppendorf, Hamberg, Germany);
- 0.2 mL PCR amplification tubes (Bio Basic Inc., Markham, ON, Canada);
- Kimwipes^®^ (Kimberly-Clark Professional, Kimtech, Roswell, GA, USA);
- Spin baskets (Eppendorf, Hamberg, Germany);
- Conical tubes (5 mL, 15 mL, or 50 mL) (Corning, Corning, NY, USA);
- Extraction Buffer (1 M Tris-HCL, 0.5 M EDTA, 20% SDS, Sodium Chloride);
- TE Buffer (1 M Tris-HCL, 0.5 M EDTA);
- Quantifiler™ Trio DNA Quantification Kit (Thermo Fisher Scientific, Waltham, MA, USA);
- MicroAmp™ Optical 96-Well Reaction Plate (Thermo Fisher Scientific, Waltham, MA, USA);
- MicroAmp™ Optical Adhesive Film (Thermo Fisher Scientific, Waltham, MA, USA);
- PowerPlex^®^ Fusion 6C System (Promega Corporation, Madison, WI, USA);
- PowerPlex^®^ Y23 System (Promega Corporation, Madison, WI, USA);
- Hi-Di™ Formamide (Thermo Fisher Scientific, Waltham, MA, USA).

For decontamination purposes:

- Deionized water (DiH_2_O);
- Bleach solution (10–20%).

### 2.2. Equipment

- EZ1^®^ Advanced XL (QIAGEN, Hilden, Germany);
- EZ1^®^ Advanced XL DNA Investigator^®^ Card (QIAGEN, Hilden, Germany);
- EZ1^®^ Advanced XL Reagent Rack (QIAGEN, Hilden, Germany);
- EZ1^®^ Advanced XL Tip Rack (QIAGEN, Hilden, Germany);
- Pipettes (2.5 µL, 10 µL, 20 µL, 100 µL, 200 µL, and/or 1000 µL);
- Vortex;
- Pulse Spinner;
- Thermomixer (56 °C and 70 °C);
- Centrifuge;
- Microcentrifuge tube rack;
- Tweezers;
- Scalpel and/or scissors;
- QuantStudio™ 5 Real-Time PCR System (Thermo Fisher Scientific, Waltham, MA, USA);
- Veriti™ Thermal Cycler (Thermo Fisher Scientific, Waltham, MA, USA);
- 3500 Genetic Analyzer (Thermo Fisher Scientific, Waltham, MA, USA).

### 2.3. Software

- HID Real-Time PCR Analysis Software v1.3 (Thermo Fisher Scientific, Waltham, MA, USA);
- GeneMapper™ ID-X v1.4 (Thermo Fisher Scientific, Waltham, MA, USA).

## 3. Procedure

### 3.1. Immunochromatographic Test and Processing [2,3]

**Figure 1:**
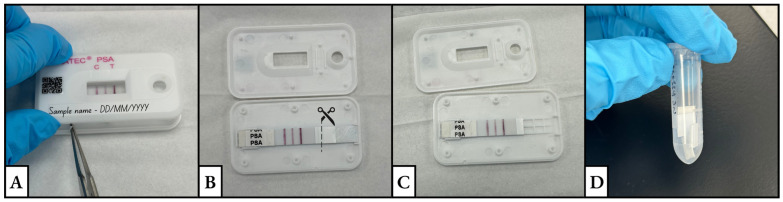
The series of images depicts the process of (**A**) opening a test cassette using tweezers, (**B**,**C**) cutting the sample pad, and (**D**) placing the cuttings in a clean and labeled tube.

1. Prepare the sample type according to the immunochromatographic test instructions.
  - Viscous samples should be diluted until the sample flows smoothly on the test membrane.
2. Cotton swabs, cloth, or condom pieces should be extracted in enough buffer. The cut piece should be between 0.25 and 1 cm^2^ in size and extracted in about 0.5–1 mL buffer.
3. PSA Semiquant Test: liquid samples diluted at least 1:500 with provided buffer prior to testing.
  - PMB Test: liquid samples diluted at least 1:50 with provided buffer prior to testing.
4. An extraction time of about 10 min is recommended. However, older and smaller stains may need a longer extraction time.
5. Add 120 µL (3 drops with the provided dropper) of the prepared sample according to the immunochromatographic test protocol.
6. Read the test result after 10 min at room temperature and let the sample pad dry out.
7. Run negative controls using 120 µL of the kit provided buffer. Prepare and run positive controls.
8. PSA Semiquant positive control: Dilute purchased seminal fluid to 1:500 with the provided buffer. Add 120 µL of dilution to the sample well and read the test result after 10 min at room temperature.
9. PMB Test positive control: Dilute purchased menstrual blood to 1:50 with the provided buffer. Add 120 µL of dilution to the sample well and read the test result after 10 min at room temperature.
10. Once dry, open the cassette along the seam using a pair of sterile metal tweezers over a clean, sterile surface (Figure 1A).
11. Using a clean scalpel or clean pair of scissors, cut off the sample pad from the rest of the test strip. Cut into multiple pieces (~5 mm). This size allows for the extraction buffer to completely cover the sample pad (Figure 1B,C).
12. Place sample pad cuttings in a clean, labeled tube. Store samples (between 2 °C and 8 °C) for further testing (Figure 1D).

### 3.2. Differential Extraction from the Sample Pad (Figure S1) [10,11]

1. Prepare a master mix (500 µL Extraction Buffer, 5 µL of Proteinase K, and 1 µL of Carrier RNA per sample) in an appropriately sized conical tube. Vortex and pulse spin. Pipette 506 µL of the master mix into each control and sample tube.
2. Vortex and briefly pulse spin. Incubate and shake at 56 °C and 850 RPM for 40 min.
3. Vortex and briefly pulse spin. Transfer the sample pad pieces to a spin basket with clean tweezers and place the spin basket back into the tube.
4. Centrifuge for 10 min at 14,000 RPM with the hinge pointing up.
5. Remove and discard the spin basket. Sample pad pieces can be appropriately repackaged and stored based on the laboratory’s procedures. The pellet will be at the bottom of the tube on the hinged side.
6. Removing as much liquid as possible, carefully transfer the supernatant without disturbing the sperm cell pellet to a new microcentrifuge tube labeled “F1”. Set aside for processing or store the “F1” tubes.
7. Pipette 500 µL of Buffer G2 into each sample/control tube (now the “F2” tubes). Vortex and centrifuge for 5 min at 14,000 RPM with the hinge pointing up.
8. Carefully remove and discard the supernatant without disturbing the pellet, as done previously.
9. Prepare a master mix (160 µL of Buffer G2, 10 µL of Proteinase K, and 40 µL DTT per sample) in an appropriately sized conical tube. Vortex and pulse spin. Pipette 210 µL into each “F2” tube.
10. Vortex and pulse spin. Incubate and shake at 70 °C and 850 RPM for 40 min.
11. Vortex and pulse spin. Cut the hinge of each tube. Do not discard the cap at this point.
12. Following manufacturer’s guidelines, process samples with the EZ1&2^®^ Advanced XL DNA Investigator^®^ Kit on the EZ1^®^ Advanced XL following the trace protocol, selecting an elution volume of 50 µL in TE Buffer.
13. Store the eluted DNA for downstream processing. The extracts should be stored in the refrigerator (between 2 °C and 8 °C) for short-term storage and in the freezer (approximately −20 °C) for long-term storage.

A graphic illustration showcases a comprehensive overview of the entire differential extraction process, portraying each key step in the procedure, which can be found in Supplementary Figure S1.

### 3.3. Downstream Processing

#### 3.3.1. DNA Quantification with Quantifiler™ Trio on Applied Biosystems QuantStudio™ 5 Real-Time PCR System [12]

14. Thaw and prepare serial dilutions of the Quantifiler™ THP DNA Standard 100 ng/μL with the Quantifiler™ THP DNA Dilution Buffer, making concentrations of 50 ng/µL, 5 ng/µL, 0.5 ng/µL, 0.05 ng/µL, and 0.005 ng/µL (Table S1).
15. Prepare a master mix for the total number of standards, NTCs, and samples. Standards and NTCs are prepared in duplicate. The master mix consists of the Quantifiler™ THP PCR Reaction Mix and Quantifiler™ Trio Primer Mix, totaling a volume of 9 µL per reaction for a half-scale reaction (Table 1).
16. Vortex the master mix and pipette 9 µL into each used well of a MicroAmp™ Optical 96-Well Reaction Plate.
17. Add 1 µL of each standard, sample, or No Template Control (NTC) to the plate. Quantifiler™ THP DNA Dilution Buffer must be used as the NTC for the run.
18. Use a MicroAmp™ Optical Adhesive Cover to seal the plate and centrifuge the plate briefly. Ensure the contents of the wells are on the bottom and that there are no air bubbles.
19. Load the plate onto the QuantStudio™ 5 according to the manufacturer’s protocol.
20. Launch the HID Real-Time PCR Analysis Software v1.3 from the desktop. Select “New Experiment” and choose the “Quantifiler™ Trio” template.
21. Names of controls and samples can be added by double clicking on an empty box and clicking the “Add New Sample” icon.
22. Save the plate file. Select the “Setup” tab in the “Experiment” menu and click “Run Method”. Ensure the sample volume is 10 µL and set up the parameters for the thermocycler: Holding Stage, 95 °C 2 min; Cycling Stage, Step 1: 95 °C 9 s, and Step 2: 60 °C 30 s for 40 cycles. The ramp rate for all steps is 2.5 °C/s.
23. On the “Run” tab, click “Start Run”. After the run has completed, click “Analyze” to analyze the data.
24. For the standard curve, acceptable R^2^ values for each target is ≥0.98. The acceptable range and average slope for each target are shown in Table S2. A slope of −3.3 indicates 100% PCR efficiency.
25. The acceptable ranges for the y-intercept values for each target are shown in Table S3.
26. The NTC should be Undetermined for the Small Autosomal, Large Autosomal, and Y Targets.
27. The acceptable value ranges for the slope, y-intercept, and R^2^ values of the standard curve were established using internal validation studies. These values will differ based on internal validation studies performed by each laboratory.
28. For proceeding to STR amplification, the concentration of the Small Autosomal target in ng/µL is used to determine the input volume.

#### 3.3.2. Amplification of STRs Using the PowerPlex^®^ Fusion 6C System and/or the PowerPlex^®^ Y23 System (Half-Volume Reaction) [13]

1. Thaw and vortex the pre-amplification component tubes and vortex before each use.
2. Calculate the required input volumes for each control/sample to achieve a DNA input target of 1 ng using the small autosomal target quantitation result. A positive control, 2800 M Control DNA, should also be included at this 1 ng DNA input target. Total volume in each tube should be 12.5 µL (master mix + input DNA + TE Buffer).
  - PowerPlex^®^ Fusion 6C: the maximum input volume of template DNA is 7.5 µL.
  - PowerPlex^®^ Y23: the maximum input volume of template DNA is 8.75 µL.
3. Prepare a master mix for the total number of samples, positive controls, and negative controls (with TE Buffer) and account for overage.
  - The PowerPlex^®^ Fusion 6C master mix consists of 2.5 µL of PowerPlex^®^ Fusion 6C 5× Master Mix and 2.5 µL of PowerPlex^®^ Fusion 6C 5× Primer Pair Mix. Vortex and add 5 µL of master mix into each autosomal STR amplification tube (0.2 mL PCR tubes).
  - The PowerPlex^®^ Y23 master mix consists of 2.5 µL of PowerPlex^®^ Y23 5× Master Mix and 1.25 µL of PowerPlex^®^ Y23 10× Primer Pair Mix. Vortex and add 3.75 µL of master mix into each Y-STR amplification tube (0.2 mL PCR tubes).
4. Prepare the positive control. Dilute 2800 M Control DNA by pipetting 1 µL of 2800 M and 9 µL of TE Buffer. Vortex the dilution and pipette 1 µL into the appropriate amplification tube containing master mix.
  - PowerPlex^®^ Fusion 6C: add 6.5 µL of TE Buffer to make the final volume 12.5 µL.
  - PowerPlex^®^ Y23: add 7.75 µL of TE Buffer to make the final volume 12.5 µL.
5. Prepare the negative control.
  - PowerPlex^®^ Fusion 6C: add 7.5 µL of TE Buffer to the appropriate amplification tube to make the final volume 12.5 µL.
  - PowerPlex^®^ Y23: add 8.75 µL of TE Buffer to the appropriate amplification tube to make the final volume 12.5 µL.
6. Add the calculated volume of each sample to their respective amplification tubes. Remaining volume must be filled with TE Buffer (final volume must be 12.5 µL). Cap the tubes.
7. Place the tubes in the Veriti™ Thermal Cycler with the following parameters:
  - PowerPlex^®^ Fusion 6C: Hold 96 °C 1 min; 29 cycles of 96 °C 5 s, and 60 °C 1 min; Hold 60 °C for 10 min; Hold 4 °C.
  - PowerPlex^®^ Y23: Hold 96 °C 2 min; 30 cycles of 94 °C 10 s, 61 °C 1 min, 72 °C for 30 s; Hold 60 °C for 20 min; Hold 4 °C.
8. After the run is complete, the samples can either be stored at −20 °C or can immediately move onwards to fragment analysis.

#### 3.3.3. Fragment Analysis on the 3500 Genetic Analyzer (Capillary Electrophoresis) [14]

1. Vortex and pulse spin the allelic ladders, controls, samples, Hi-Di™ formamide, and ILS size standards.
  - Two allelic ladders are included for each kit for interpretation of bins.
2. Prepare a MicroAmp™ Optical 96-Well Reaction Plate.
3. Prepare a loading master mix of Hi-Di™ formamide and the ILS size standards considering the total number of samples, controls, and ladders and account for overage. Vortex and briefly pulse spin before pipetting 10 µL into the respective wells.
  - PowerPlex^®^ Fusion 6C: pipette 13.2 µL of WEN ILS 500 size standard into a 1.5 mL tube with 250 µL of Hi-Di™ formamide.
  - PowerPlex^®^ Y23: pipette 13.2 µL of WEN ILS 500 Y23 size standard into a 250 µL tube of Hi-Di™ formamide.
4. Pipette 1 µL of each control, sample, and appropriate kit allelic ladder into the respective wells (PowerPlex^®^ Fusion 6C Allelic Ladder Mix or PowerPlex^®^ Y23 Allelic Ladder Mix).
5. Cover the plate using a 96-well plate septa. Vortex and centrifuge the plate to draw liquid to the bottom of the well and remove any air bubbles.
6. Place the plate into the 3500 plate base and plate retainer and close so that the holes of the plate retainer and septum are aligned. Place the plate in the autosampler of the 3500.
7. Create a new plate file in the instrument. The plate properties are as follows: 96 wells, HID plate type, 36 cm capillary length, POP4 polymer.
8. Select the appropriate cells and assign the applicable assays with the appropriate run parameters based on internal validation studies. Please note that the following injection times were followed: 12 s for PowerPlex^®^ Fusion 6C and 3 s for PowerPlex^®^ Y23.
9. Analyze the data using GeneMapper™ ID-X or another applicable genotyping software program.

## 4. Materials and Methods

Menstrual blood samples were collected from three female volunteers. Each volunteer provided a sample from each day of their menstrual cycle by depositing the collected liquid into a provided conical tube. The samples were labeled with the corresponding day of the cycle and stored in a refrigerator at 2 °C until needed. Seminal fluid samples, containing sperm cells, were self-collected from three male volunteers into provided specimen cups. Seminal fluid samples were frozen at −18 °C until required. Additionally, each volunteer provided a buccal swab for reference DNA. All volunteers were at least 18 years old. This study was approved by the Arcadia University Institutional Review Board (IRBNet ID: 2061027-3).

Mixtures were prepared for all nine possible pairings of menstrual blood and seminal fluid samples in the following proportions (Female to Male): 100 to 1 (200 μL menstrual blood, 2 μL of seminal fluid), 10 to 1 (50 μL menstrual blood, 5 μL of seminal fluid), and 1 to 1 (equal volumes). Additionally, two mixtures with 50 to 1 proportion were prepared (100 μL of menstrual blood, 2 μL of seminal fluid). The 1 to 1 and 50 to 1 mixtures were prepared for select pairs due to limited sample volume. Each mixture was applied to each LFI test in duplicate, following the “Immunochromatographic Test and Processing” procedure outlined above. As described in the procedure, the mixtures were diluted with the respective LFI test kit buffers to the stated recommended dilutions of 1:50 for the PMB Test and 1:500 for the PSA Semiquant prior to being tested. After interpretation of the test results, the sample pads from the LFI tests were cut and processed also according to the procedure described above. Additionally, positive (pad fragment with no mixed sample) and negative controls (pad fragment without sample) for both the PSA Semiquant and PMB Test were prepared and run through extraction, quantification, and STR typing utilizing capillary electrophoresis.

## 5. Expected Results

All mixtures applied to the LFI tests yielded a positive result for the presence of the associated fluid. All positive and negative controls for the PSA Semiquant and PMB Tests performed as expected. Additionally, all downstream processing controls also performed as expected.

### 5.1. DNA Concentration

**Figure 2:**
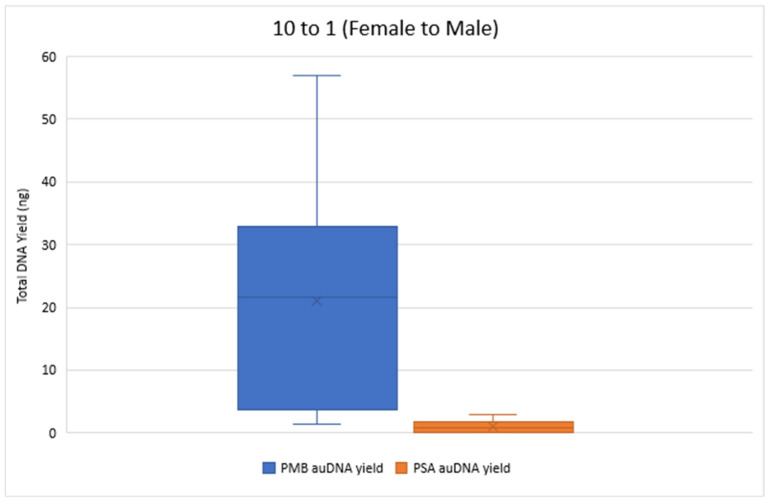
Total autosomal DNA (auDNA) Yield (ng) from 10 to 1 Sample Pads. The total auDNA yield from the PMB tests ranged from 1.36 ng to 56.89 ng, whereas the yield from the PSA tests ranged from 0.042 ng to 2.84 ng.

**Figure 3:**
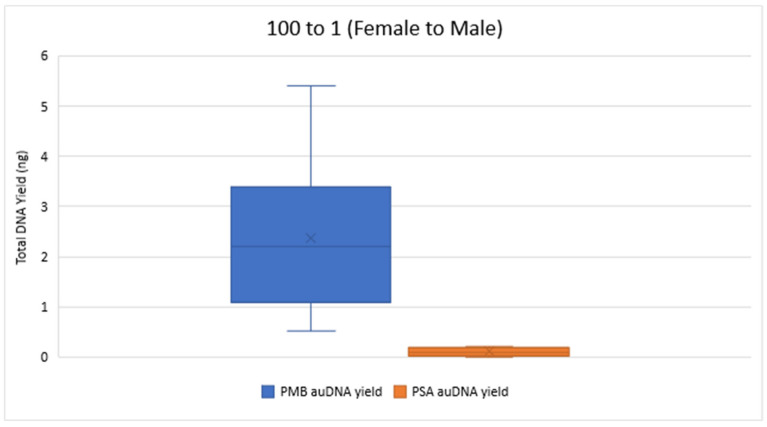
Total autosomal DNA (auDNA) Yield (ng) from 100 to 1 Sample Pads. The total auDNA yield from the PMB tests varied from 0.51 ng to 5.40 ng, whereas the yield from the PSA tests ranged from 0 ng to 0.22 ng.

Figure 2 and Figure 3 compare the total DNA yield (ng) for 10 to 1 and 100 to 1 Female to Male samples from both PMB and PSA tests, with all samples quantified in duplicate. The results show a wide range of total DNA yield across these proportions from the PMB tests. The 50 to 1 PMB samples had significantly lower DNA yields (0.019 ng–0.124 ng) for autosomal DNA and 0.014 ng–0.121 ng for Y chromosome DNA compared to the 10 to 1 and 100 to 1 proportions. In the 1 to 1 samples the PMB test yielded much higher DNA yields (242.88 ng for autosomal DNA and 253.24 ng for Y chromosome DNA) than the PSA Semiquant test (15.11 ng and 16.51 ng, respectively).

The total Y-DNA yield for the 10 to 1 mixture proportions ranged from 0.86 ng to 49.68 ng for PMB and from 0.04 ng to 3.05 ng for PSA. For the 100 to 1 proportion, total Y-DNA yielded values ranging from 0.30 ng to 4.78 ng for PMB and 0 ng to 0.61 ng for PSA. Overall, the extracts from the PMB test had a greater quantity of DNA than the PSA test across the proportions tested. Table 2 showcases the ranges of the IPC_CT_ values and the degradation indexes for the 10 to 1 and the 100 to 1 mixture proportions for both LFI tests. All IPC_CT_ values were within range, and no IPC_CT_ flags were triggered. These values indicate that there were no signs of PCR inhibitor presence. The degradation index ranges indicate that the extracted DNA was at most slightly to moderately degraded.

### 5.2. STR Profiles

For the autosomal STR results, all 51 male donor alleles were detected from all nine of the 10 to 1 mixture proportions and in eleven of the thirteen 100 to 1 mixtures from the PMB tests. One 100 to 1 mixture yielded no profile, and another produced a partial profile with 33 of the 51 male donor alleles. Of the nine 10 to 1 PMB profiles, peak height imbalance of some form was observed in five of the electropherograms, and a few minor female alleles were noted in six of the electropherograms. Ten of the thirteen 100 to 1 PMB STR profiles had peak height imbalance to varying degrees and there was presence of female alleles in the electropherograms. Two of the three 10 to 1 mixtures from the PSA Semiquant tests recovered all male donor alleles. The two 10 to 1 PSA profiles did display peak height imbalance at a few loci, and there were some minor female alleles present within the electropherogram. The 50 to 1 mixture from the PMB test produced a partial profile with 13 of 51 alleles. There was evidence of dropout and alleles that failed to meet the analytical threshold, and thus failed to be called. Both 1 to 1 mixtures from the PMB test and PSA Semiquant resulted in all male donor alleles detected. The 1 to 1 PMB profile showed peak height imbalance at one locus. See Figures S2–S4 for example autosomal STR profiles from the 100 to 1 proportions.

As for the Y-STR results, all 24 male donor alleles were obtained from nine of the 10 to 1 mixtures and ten of twelve 100 to 1 mixtures from the PMB tests, with one mixture yielding no profile, and another a partial profile with 15 of 24 alleles. Four of the nine 10 to 1 PMB Y-STR profiles showed signs of peak height imbalance, but the 100 to 1 PMB Y-STR profiles showed no signs of peak height imbalance. The partial profile had allelic dropout and peakspresent on the electropherogram that were below the analytical threshold, and therefore failed to be called. For the PSA Semiquant tests, all male donor alleles were detected from two of the three 10 to 1 mixtures and there was no peak height imbalance. Both 1 to 1 mixtures from the PMB and PSA Semiquant tests detected all male donor alleles, and the electropherograms showed no signs of peak height imbalance. See Figures S5 and S6 for example Y-STR profiles from the 100 to 1 proportions.

## 6. Conclusions

The protocol described has been rigorously tested in experiments, including those by Neilson et al., with the results highlighted above. When the sample pads of these LFI tests were extracted following this protocol, male donor alleles (autosomal and Y-STR) were detected from the PMB sample pads and the PSA sample pads, as indicated previously [15]. When utilizing this differential extraction protocol on sample pads, full male profiles were able to be obtained from the 10 to 1 and 100 to 1 mixtures of menstrual blood and semen deposited onto the PMB Test. With PSA Semiquant sample pad as the substrate, full male profiles were only obtained from the 10 to 1 proportion. This research has indicated that even with proportions of 100 to 1 (F to M), a full STR profile of the male can be obtained for both autosomal and Y-STRs when extracting from the PMB Test sample pad. This method effectively separates sperm DNA from non-sperm DNA in mixed samples by selectively lysing epithelial cells first, while preserving sperm heads, followed by targeted lysis of the sperm fraction. These findings align with established differential extraction techniques commonly used in forensic casework, where sequential lysis ensures the enrichment of distinct DNA fractions with minimal cross-contamination, enabling reliable analysis of complex biological mixtures [16,17].

In Neilson et al. [15], the PMB Test consistently resulted in extracts with higher concentrations of DNA compared to the PSA Semiquant. The observed variations between the tests may be due to differences in test dilutions, buffer components, DNA concentrations, and uneven distribution of cellular components during preparation and manual pipetting. A limitation to consider is that the PSA Semiquant, the LFI for presumptive seminal fluid detection, did not yield similar quantities of DNA as the PMB Test. For many of the proportions tested, without extracting from the PMB Test, a male profile would not have been obtained. Future work could investigate possible ways to increase the concentration of DNA obtained from the PSA Semiquant, such as concentrating the extracts and/or utilizing the entire test strip, like reported in Conte et al. (2022), or extracting from the remaining PSA Semiquant buffer solution, as reported in Barbaro et al. (2015) and Zapico and Roca (2024), and should be investigated [6,8,18]. Additionally, liquid mixtures were utilized in the above study; however, more common case-type samples are swabs and other substrates. Further studies could be conducted utilizing mixtures of these body fluids deposited onto swabs to determine if similar concentrations can be obtained. Future studies could also compare traditional DNA extraction methods and the protocol displayed here.

When samples are collected as evidence, other contaminants may be present and could impact the extraction process and the overall DNA quality. However, the EZ1^®^ Advanced XL utilizes magnetic particles, which bind DNA, while several washes remove all PCR inhibitors [19]. Following this differential extraction protocol and properly operating the EZ1^®^ Advanced XL should limit the factors that may have an impact on extraction quality. The results above can attest to this, as there were no signs of PCR inhibition or contamination in the samples (See Figures S2–S6 for example electropherograms). Overall, this differential extraction method from immunochromatographic pads has proven effective in recovering both autosomal and Y-STR DNA from casework-type mixture body fluid samples (i.e., semen and menstrual blood), thus demonstrating the potential of utilizing this procedure in sexual assault cases [15]. This protocol is valuable when minimal evidence remains after immunochromatographic tests, and can be applied to other such tests or integrated with robotic purification platforms. Additionally, DNA can be efficiently recovered from sample pads, and potentially applied to a wide range of applications beyond forensics.

## Figures and Tables

**Table 1:** Quantifiler™ Trio components per half-scale reaction.

**Reaction Component**	**Volume per Reaction**
Quantifiler™ THP PCR Reaction Mix	5 µL
Quantifiler™ Trio Primer Mix	4 µL
Final Volume	9 µL

**Table 2:** Quantifiler™ Trio IPC_CT_ and degradation index values for the 100 to 1 and 10 to 1 mixture proportions.

Proportion (F to M)	LFI Test	IPC_CT_ Value Range	Degradation Index Range
10 to 1	PMB Test	27.70–27.95	0.72–1.07
	PSA Semiquant	27.80–27.86	0.82–0.99
100 to 1	PMB Test	27.05–28.88	0.58–2.16
	PSA Semiquant	28.37	N/A

## Data Availability

Data is available upon request.

## Supplementary Materials

The following supporting information can be downloaded at: https://www.mdpi.com/article/10.3390/mps8010002/s1. Figure S1. This graphic illustrates a comprehensive overview of the entire differential extraction process, portraying each key step in the procedure. Table S1: Quantifiler™ Trio standard preparation. Table S2. Slope acceptable ranges and averages for each Quantifiler™ Trio target. Table S3. Y-intercept acceptable ranges for each Quantifiler™ Trio target. Figure S2. Electropherogram from sample F1M2 100 to 1 (PMB), displaying a high-quality STR result with clear, well-defined peaks and no evidence of degradation or artifacts. Figure S3. Electropherogram from sample F1M4 100 to 1 (PMB), illustrating a mid-quality STR result. Figure S4. Electropherogram from sample F3M3 100 to 1 (PMB), showing a low-quality STR result. Several alleles have dropped out, and there is noticeable peak height imbalance at multiple loci. Figure S5. Electropherogram from sample F1M2 100 to 1 (PMB), showing a high-quality Y-STR result. All peaks exceed both the analytical and stochastic thresholds. The peaks are sharp, well-resolved, and exhibit minimal stutter, all within the acceptable range. Furthermore, there is no peak height imbalance, especially evident at the DYS385 locus. Figure S6. Electropherogram from sample F3M3 100 to 1 (PMB), illustrating a low-quality Y-STR result. Several peaks exhibit visible dropout, failing to meet the analytical threshold and therefore not being called. Additionally, most of the called peaks do not reach the stochastic threshold.
