# Supplementary material for: Differential DNA Extraction from Lateral Flow Immunochromatographic Tests via the EZ1® Advanced XL System
Source: Methods Protoc. 2025 Jan 2;8(1):2. doi: 10.3390/mps8010002 (PMC11755612; doi:10.3390/mps8010002)
Supplement: Supplementary file 1 [file mps-08-00002-s001.zip › mps-3346232-supplementary.pdf]

Supplemental Material

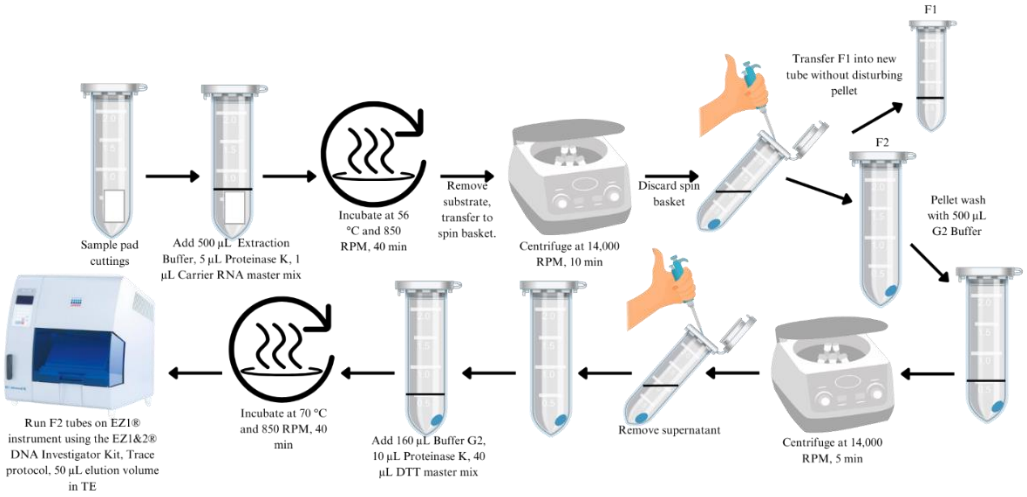

Figure S1. This graphic illustrates a comprehensive overview of the entire differential extraction process, portraying each key step in the procedure.

Table S1. Quantifiler™ Trio standard preparation.

Standard Concentration	Volume of DNA Added	Volume of Quantifiler™ THP DNA Dilution Buffer
50 ng/µL	5 µL from Quantifiler™ THP DNA Standard (100 ng/µL)	5 µL
5 ng/µL	5 µL from 50 ng/µL dilution	45 µL
0.5 ng/µL	5 µL from 5 ng/µL dilution	45 µL
0.05 ng/µL	5 µL from 0.5 ng/µL dilution	45 µL
0.005 ng/µL	5 µL from 0.05 ng/µL dilution	45 µL

Table S2. Slope acceptable ranges and averages for each Quantifiler™ Trio target.

Quantifiler™ Trio target	Slope Range	Average slope
Small Autosomal	–3.0 to –3.6	-3.3
Large Autosomal	–3.1 to –3.7	-3.4
Y Target	–3.0 to –3.6	-3.3

Table S3. Y-intercept acceptable ranges for each Quantifiler™ Trio target.

Target	Acceptable Y-Intercept Range
Small Autosomal	25.29 – 27.91
Large Autosomal	24.45 – 25.82
Y Target	24.85 – 26.84

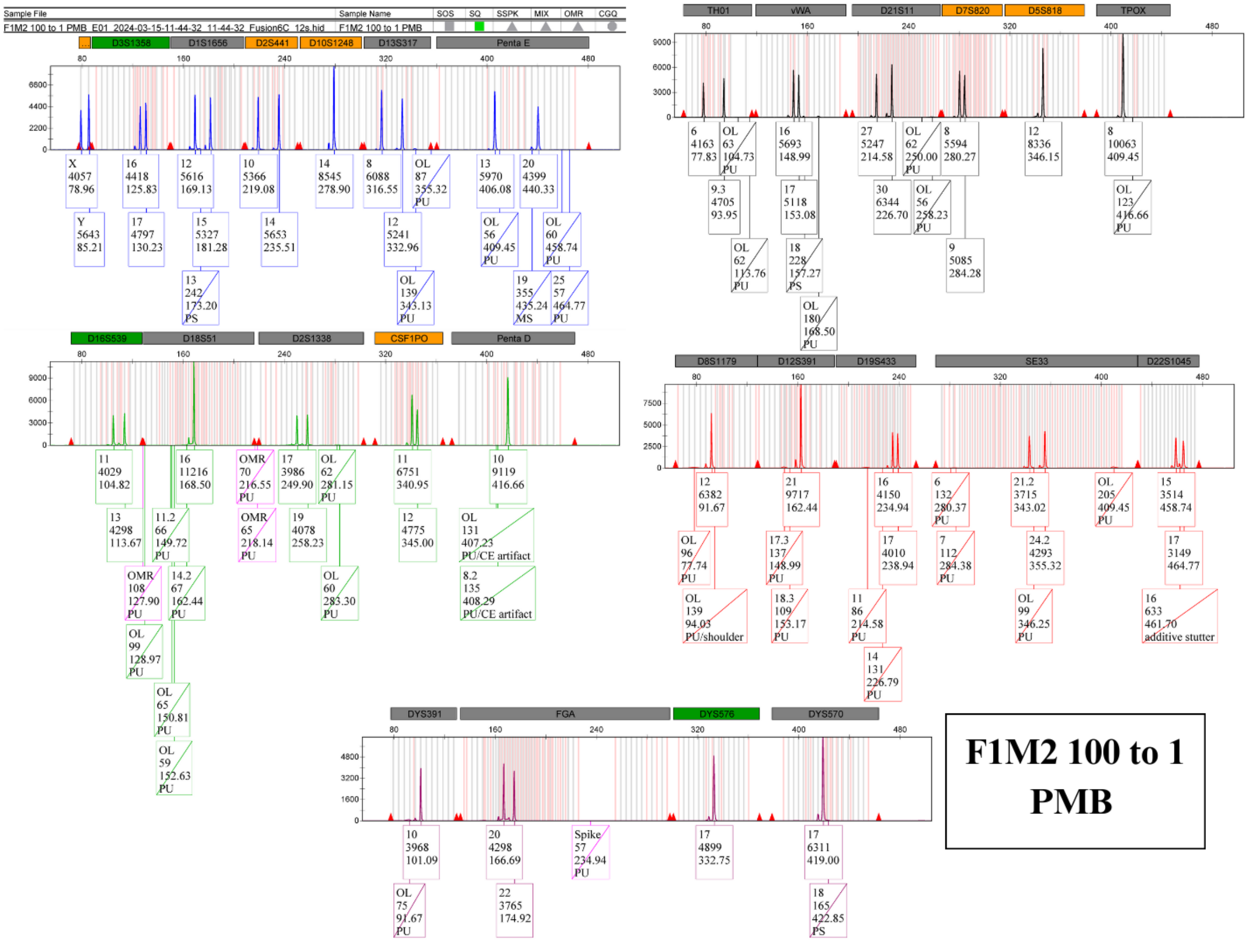

Figure S2. Electropherogram from sample F1M2 100 to 1 (PMB), displaying a high-quality STR result with clear, well-defined peaks and no evidence of degradation or artifacts.

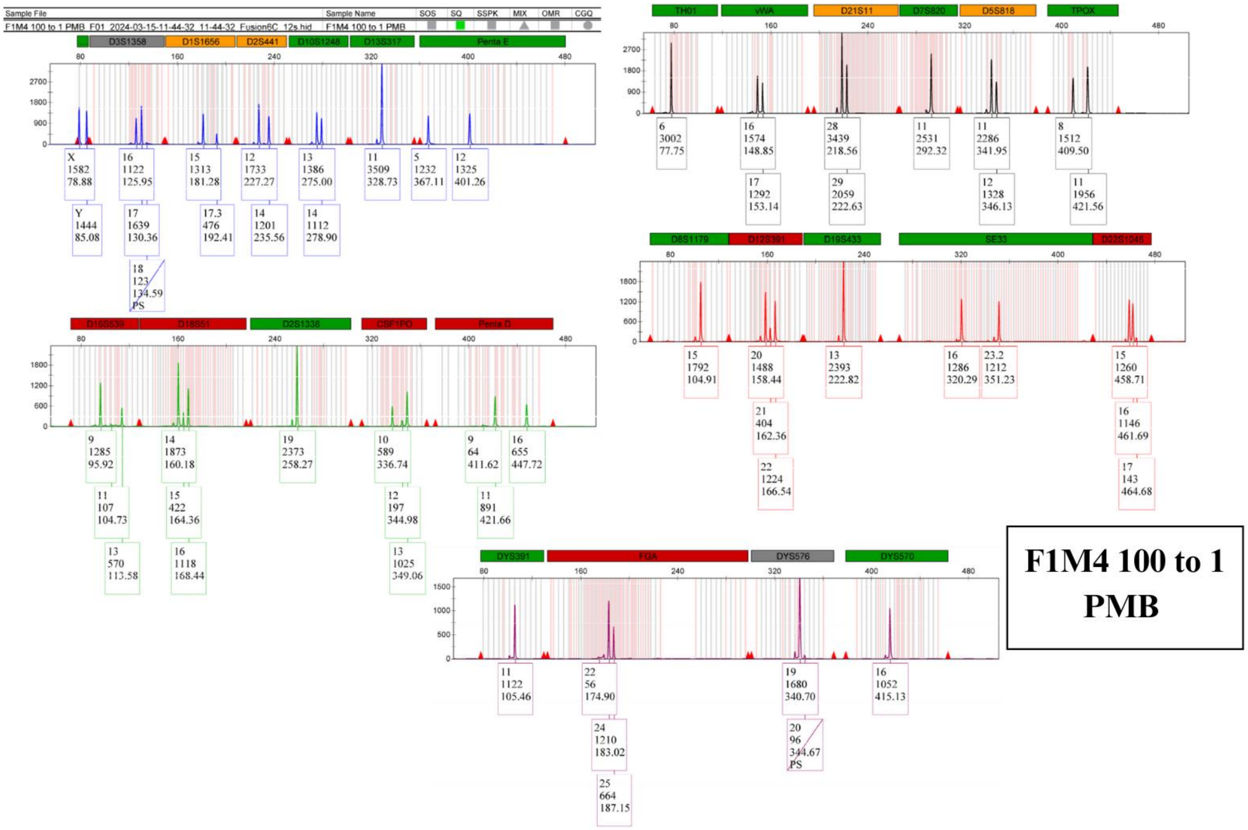

Figure S3. Electropherogram from sample F1M4 100 to 1 (PMB), illustrating a mid-quality STR result.

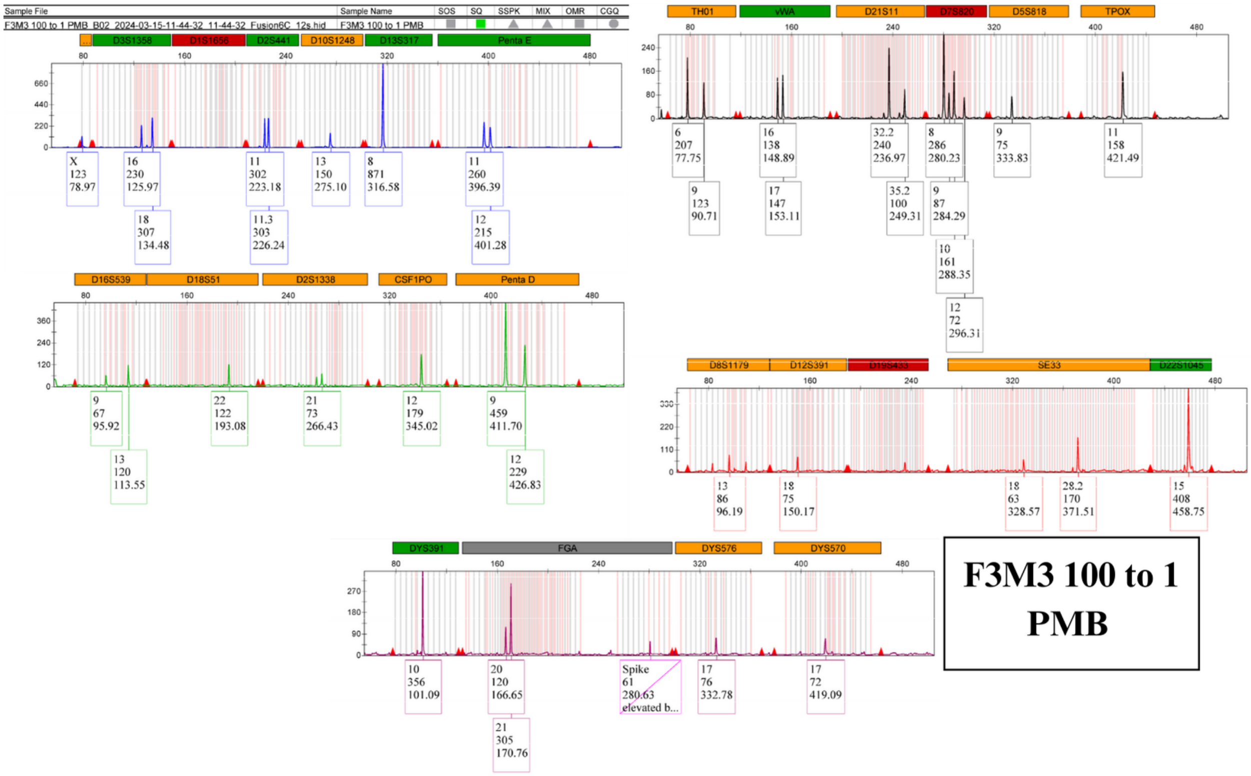

Figure S4. Electropherogram from sample F3M3 100 to 1 (PMB), showing a low-quality STR result. Several alleles have dropped out, and there is noticeable peak height imbalance at multiple loci.

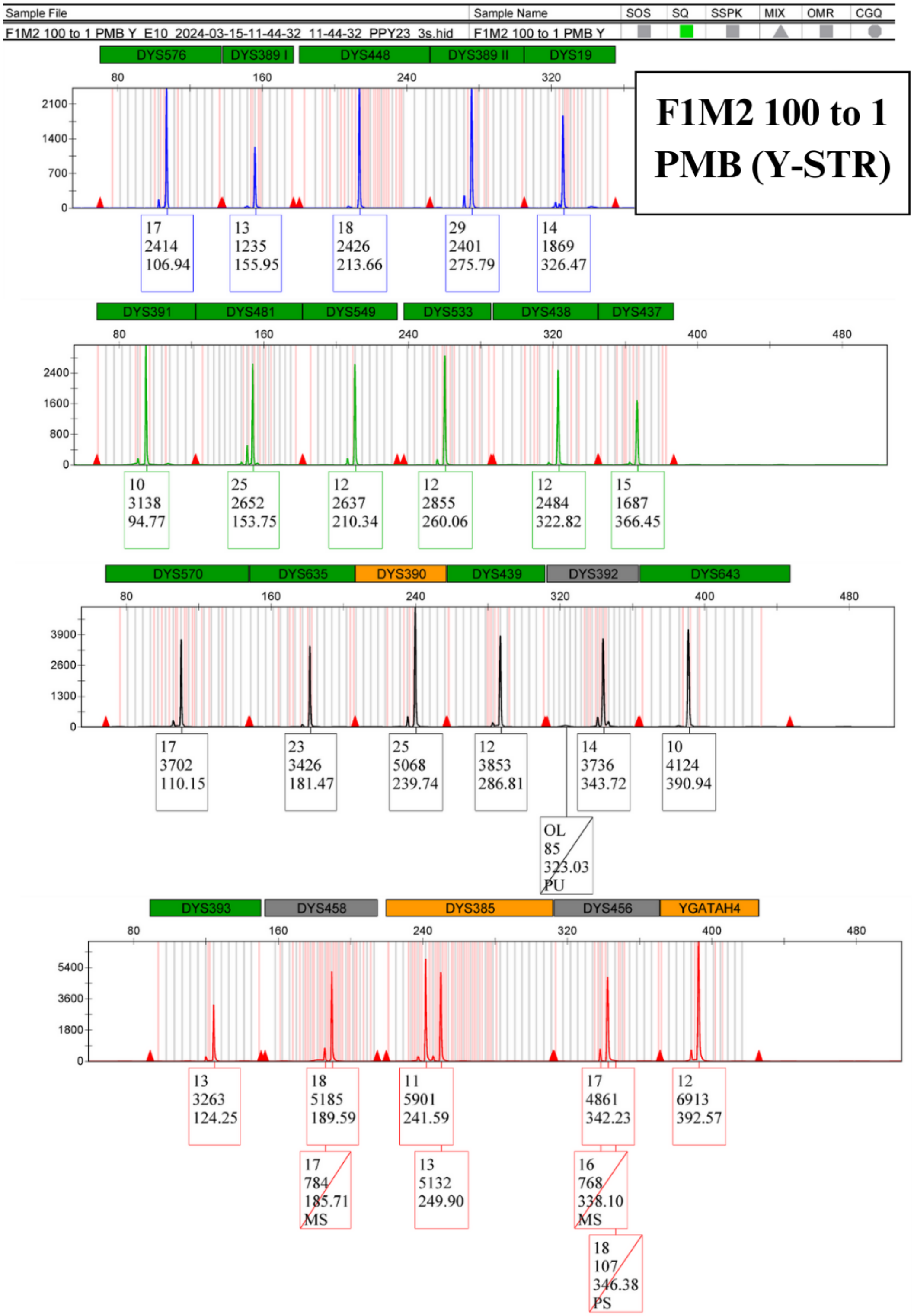

Figure S5. Electropherogram from sample F1M2 100 to 1 (PMB), showing a high-quality Y-STR result. All peaks exceed both the analytical and stochastic thresholds. The peaks are sharp, well-resolved, and exhibit minimal stutter, all within the acceptable range. Furthermore, there is no peak height imbalance, especially evident at the DYS385 locus.

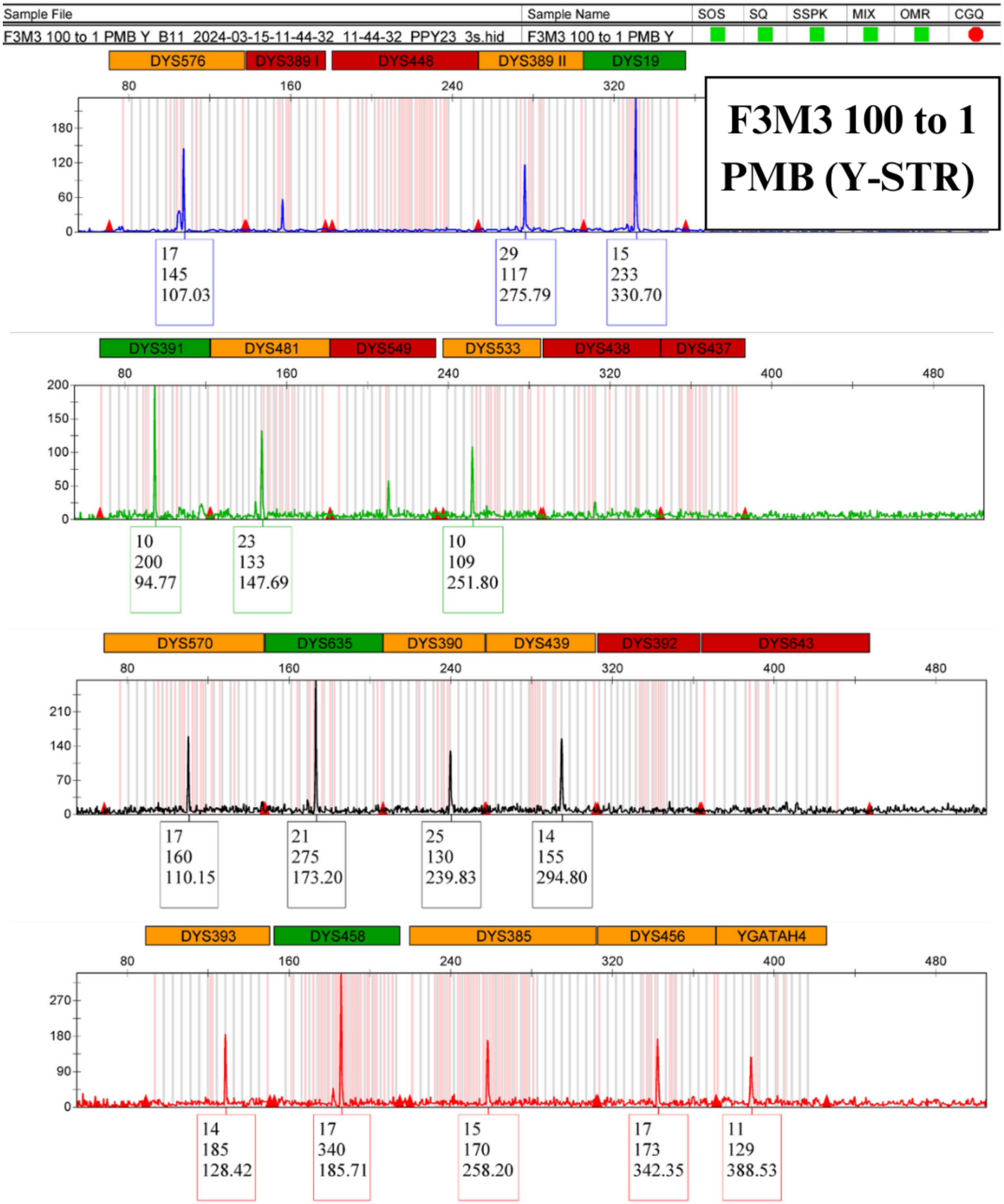

Figure S6. Electropherogram from sample F3M3 100 to 1 (PMB), illustrating a low-quality Y-STR result. Several peaks exhibit visible dropout, failing to meet the analytical threshold and therefore not being called. Additionally, most of the called peaks do not reach the stochastic threshold.
